# Interferon Alpha Association with Neuromyelitis Optica

**DOI:** 10.1155/2013/713519

**Published:** 2013-11-18

**Authors:** Nasrin Asgari, Anne Voss, Troels Steenstrup, Kirsten Ohm Kyvik, Egon Stenager, Soeren Thue Lillevang

**Affiliations:** ^1^Department of Neurology, Vejle Hospital, 7100 Vejle, Denmark; ^2^Institute of Molecular Medicine, University of Southern Denmark, J.B. Winsloewsvej 19,3, 5000 Odense C, Denmark; ^3^Institute of Regional Health Research, University of Southern Denmark, 29 Sdr. Boulevard, 5000 Odense C, Denmark; ^4^Department of Rheumatology, Odense University Hospital, Winsloewsvej 19B, 2., 5000 Odense C, Denmark; ^5^Department of Biostatistics, University of Southern Denmark, 29 Sdr. Boulevard, 5000 Odense C, Denmark; ^6^Odense Patient Data Explorative Network, Odense University Hospital, Soenderjyllands, Hospital, Egelund 10, 6200 Aabenraa, Denmark; ^7^The Multiple Sclerosis Clinic of Southern Jutland, Vejle, Esbjerg, Soenderborg, Denmark; ^8^Department of Neurology, Sønderborg Hospital, Denmark; ^9^Department of Clinical Immunology, Odense University Hospital, Denmark

## Abstract

Interferon-alpha (IFN-**α**) has immunoregulatory functions in autoimmune inflammatory diseases. The goal of this study was to determine occurrence and clinical consequences of IFN-**α** in neuromyelitis optica (NMO) patients. Thirty-six NMO and 41 multiple sclerosis (MS) patients from a population-based retrospective case series were included. Expanded Disability Status Scale (EDSS) score and MRI findings determined disease activity. Linear regression was used to assess the effects of the level of IFN-**α** on disability (EDSS). IFN-**α** was determined by sensitive ELISA assays. IFN-**α** was detectable in sera from 9/36 NMO patients, significantly more often than in the MS group (2/41) (*P* = 0.0197). A higher frequency of IFN-**α** was observed in NMO patients with acute relapse compared to NMO patients in remission (*P* < 0.001) and compared to the MS patients with relapse (*P* = 0.010). In NMO patients, the levels of IFN-**α** were significantly associated with EDSS (*P* = 0.0062). It may be concluded that IFN-**α** was detectable in a subgroup of NMO patients. Association of IFN-**α** levels with clinical disease activity and severity suggests a role for IFN-**α** in disease perpetuation and may provide a plausible explanation for a negative effect of IFN-1 treatment in NMO patients.

## 1. Introduction

Inflammation in the central nervous system (CNS) is a decisive feature of multiple sclerosis (MS) and neuromyelitis optica (NMO) [1, 2]. MS seems to be induced by T-cell-mediated attacks on the myelin, whereas NMO involves antibodies directed against the water channel aquaporin-4 (AQP4), which is highly expressed in astrocytes in the CNS [1, 3]. Immunoglobulin G (IgG) anti-AQP4 antibody (NMO-IgG) is a serum biomarker for NMO [3] and evidence from human and experimental studies indicates that anti-AQP4 antibodies/NMO-IgG are involved in the pathogenesis of NMO [4]. Other immune mechanisms may be concurrently active in NMO, notably innate immune mechanisms such as interferon (IFN) release [5]. However, the exact importance of IFNs in NMO disease pathogenesis has not yet been elucidated. Type I IFNs (IFN-1) including IFN-alpha (IFN-*α*) and IFN-beta (IFN-*β*) constitute a group of cytokines that in addition to their antiviral and antitumor immune response exert a regulatory function in autoimmune/inflammatory diseases in CNS [6]. In MS, IFN-1 is considered immunomodulatory, and recombinant IFN-*β* is standard therapy for relapsing-remitting MS [6]. The therapeutic action of IFN-*β* in MS reduces relapses and delays disability progression involving numerous mechanisms [7]. In conformity with this observation, mice deficient in IFN-1 receptor (IFNAR) signaling develop more severe experimental autoimmune encephalomyelitis (EAE) as a model for MS [8, 9]. In EAE studies, endogenous IFN-1 is expressed and acts locally to suppress inflammation as activation of a homeostatic mechanism, which downregulates EAE [8, 9]. Furthermore, recombinant IFN-1 administration can suppress EAE [8, 9]. Thus, IFN-1 signaling seems to be acting as an anti-inflammatory response in MS.

Whether IFN-1 signaling has a role in the development of NMO is unknown. Several clinical trials of IFN-*β* therapy for NMO patients have reported that, unlike MS, IFN-*β* appears to be ineffective in preventing NMO relapse and may even increase the relapse rate [10, 11]. Such differences in therapeutic response likely reflect differences between the biological disease mechanisms involved in NMO and MS. Recently, our group in an experimental mouse model of NMO showed that NMO-like lesions were remarkably reduced in mice deficient in IFNAR signaling [12]. This finding suggests that IFN-1 contributes to NMO pathogenesis as a proinflammatory cytokine, which would explain failure of IFN-*β* therapy in NMO [12]. However, the activation of IFN-1 release has not been clarified in detail in NMO patients. The aim of the present study was to investigate whether inflammatory cytokine IFN-1 detection is associated with clinical features and anti-AQP4-antibody findings in NMO.

## 2. Material and Methods

### 2.1. Study Design

A clinical database was established for NMO patients diagnosed in the time period 1998–2008 in the Region of Southern Denmark as part of a population-based study, a retrospective case series with longitudinal prospective followup [13]. NMO patients were diagnosed according to the Wingerchuk 2006 criteria [14]. Information was obtained by means of review of medical records, a questionnaire, a clinical examination, reevaluation of previous magnetic resonance imaging (MRI) of CNS, and supplementary MRIs.

### 2.2. Patients

Patients and controls in this study originated from a population-based Caucasian cohort as reported previously [15]. A total of 36 patients with definite NMO were identified in the database. All had a relapsing-remitting course except one. The female: male ratio was 2.8: 1 and mean age at onset was 35.6 years (15–64 years). A number of NMO patients up to five years preceding the NMO diagnosis received treatment on the suspicion of MS, including natalizumab in 15 patients and interferon-beta in six patients. In addition, azathioprine was given to five NMO patients and rituximab to one NMO patient at the time of diagnosis [13]. A total of 28 NMO patients were in remission and eight had acute relapse (attacks) at the time of investigation. The clinical presentation included optic neuritis (ON), transverse myelitis (TM), longitudinally extensive TM, and brainstem syndromes (Table 1).

A group of 41 patients with MS, who were identified in the same cohort, were examined clinically and radiologically verifying the diagnosis of MS [16, 17] and were used as disease controls. A total of 27 MS patients received interferon-beta and nine natalizumab. Seven MS patients had acute relapse at the time of investigation. Disease severity/disability was measured by Expanded Disability Status Scale (EDSS) score [18] as retrieved from the medical records. In case of relapse, a new score was performed. Clinical relapse was defined either by an increase of EDSS or by findings on a new MRI with gadolinium enhancement or T2-weighted lesions. In addition, 24 patients with systemic lupus erythematosus (SLE) were used as positive disease controls. No NMO or MS patients had a diagnosis of SLE.

### 2.3. Determination of Type I IFN

IFN-*α* was determined by a sensitive ELISA assay (VeriKine, assay range 9–1000 pg/mL) based on the NIH international reference standard, with no cross-reactivity with other human type I IFNs. IFN-*β* was measured by a sensitive ELISA assay (VeriKine-HS, assay range 2–150 pg/mL) with recombinant human IFN-*β* as standard. The assay showed no cross-reactivity with human IFN-*α*.

Intraassay coefficient of variation as a measure of assay variability was below 6% for standard curve points as well as patient samples.

Whole blood was collected in dry tubes (without anticoagulant), separated by centrifugation, and serum was frozen at below −25°C within 12 hours. Serum from all patients had been exposed to one previous freeze-thaw cycle.

### 2.4. Determination of AQP4 Antibody

IgG AQP4 antibodies were measured as described previously with an immunofluorescence assay using HEK293 cells transfected with recombinant human full-length *AQP4* gene (Euroimmun, Lübeck, Germany). Patient sera were screened at a 1: 10 dilution [15, 19]. No samples had been subjected to freeze-thaw cycles prior to antibody determination.

### 2.5. Standard Protocol Approvals, Registrations, and Patient Consent

The study was approved by the Committee on Biomedical Research Ethics for the Region of Southern Denmark (reference numbers S-20080142 and S-20120182) and the Danish Data Protection Agency (reference number 2008-41-2826). All patients provided written informed consent.

### 2.6. Statistics

*P* values were estimated using Fisher's exact test. The 95% confidence intervals (CI) for the odds ratios (ORs) are exact. A censored regression model (Tobit model) was used to estimate linear regression. Statistical analyses were performed using Stata 11 (StataCorp LP, College Station, TX, USA). A level of *P* < 0.05 was used as limit of significance.

## 3. Results

### 3.1. Frequencies of Elevated IFN-*α* in NMO, MS, and SLE Patients

**Figure 1:**
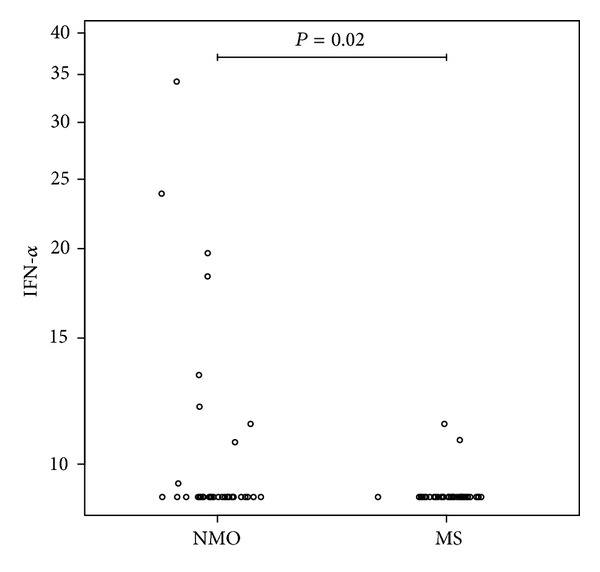
Interferon-alpha in neuromyelitis optica (NMO) and multiple sclerosis (MS). Levels of interferon-alpha in serum (pg/mL) of patients with neuromyelitis optica (NMO) and multiple sclerosis (MS). A total of 7/9 NMO patients had acute clinical attack.

Clinical and serological characteristics for NMO and MS patents are depicted in Tables 1 and 2. IFN-*α* was detected in sera from 9/36 NMO patients (5 anti-AQP4 antibody positive), significantly more than 2/41 MS patients (2/41) (odds ratio (OR) = 6.5; 95% confidence interval (CI) (1.18–64.96, *P* = 0.02)) (Figure 1). None of patients had signs of systemic viral infections or malignancies at the time of investigation. A higher frequency of detectable IFN-*α* in serum was observed in the SLE patients (16/24) compared to NMO, OR = 6; 95% CI (1.69–21.87, *P* = 0.0029) and compared to the MS group, OR = 39; 95% CI (6.60–381.47, *P* < 0.0001).

A total of 7/9 NMO patients with detectable IFN-*α* had attacks: two restricted to the brainstem, one with ON, two with TM less than three vertebral segments at the time of investigation, and two patients with LETM. A significant association was observed between the presence of IFN-*α* and acute attacks in the NMO group (*P* < 0.001), OR = 91 (5.54; 4240).

Brain MRI at disease onset demonstrated in NMO patients with detectable IFN-*α* that three (two anti-AQP4 seropositive) were normal and six (three anti-AQP4 seropositive) had nonspecific abnormalities. At followup, a total of five NMO patients with IFN-*α* had brain MRI that fulfilled the Barkhof criteria [17] for dissemination in space used in the McDonald criteria for MS [16]. Of those patients, four were AQP4 antibody positive. Four patients (one being anti-AQP4 seropositive) had nonspecific abnormalities at followup. Lesions were observed in the brainstem in six patients. Spinal cord MRI demonstrated LETM in seven NMO patients with detectable IFN-*α* (five being anti-AQP4 seropositive). Recurrent LETM was observed in three patients (two being anti-AQP4 seropositive). Spinal cord atrophy at the site of previous inflammation was seen in three patients (two being anti-AQP4 seropositive). Additionally, three NMO patients (all anti-AQP4 seropositive) had severe general atrophy of the spinal cord.

NMO patients with detectable IFN-*α* had high EDSS scores, median 7.5 (range 5–9) compared to the rest of the NMO patients (*P* < 0.0001), OR = 43.75 (3.90–602).

In the MS group 2/41 had detectable IFN-*α* and one had attack with TM (*P* = 0.32; OR = 5.5 (0.060; 444)). Brain MRI fulfilled the MS radiological criteria already at disease onset for both patients. One had a spinal cord lesion as a TM. Median EDSS score was 4.5 (range 4-5). None of the MS patients had additional autoimmune disease.

By direct comparison, the NMO group with attacks had a higher frequency of detectable IFN-*α* compared to the MS group with attacks (*P* = 0.010).

### 3.2. Frequencies of Elevated IFN-*β* in NMO, MS, and SLE Patients

The frequencies of detectable serum IFN-*β* were similar in the NMO (9/36) (5 being anti-AQP4 antibody positive) and MS (10/41) patient groups. One patient had detectable IFN-*β* as well as IFN-*α* and had acute relapse with LETM. No SLE patients had measurable serum IFN-*β*. A total of five NMO patients with detectable IFN-*β* were on IFN-*β* treatment at the time of investigation and four patients had received IFN-*β* more than 5 years before the study. The median EDSS for NMO patients with detectable IFN-*β* was 5.0 (range 2–7). In the MS group, all 10 patients were on IFN-*β* treatment with a median EDSS of 3.0 (range 2–7); one of these had acute relapse with ON at the time of investigation.

### 3.3. Levels of IFN-*α* and IFN-*β* in NMO, MS, and SLE Patients

**Figure 2:**
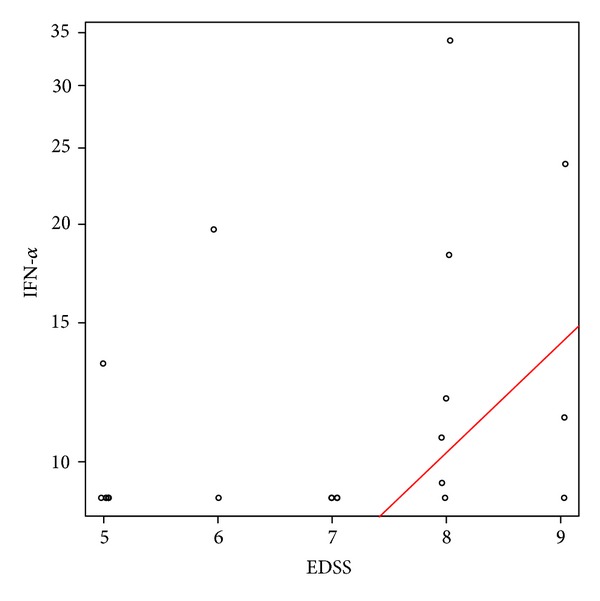
Association of interferon-alpha levels with disability in neuromyelitis optica (NMO) patients. Disability was assessed by the Expanded Disability Status Scale (EDSS) score. EDSS increased by 1 when IFN-*α* increased by 38%, *P* = 0.0062.

The levels of IFN-*α* were highest in SLE patients (9.4–118 pg/mL), followed by NMO (9.4–34 pg/mL), and were low in the MS patients (11 pg/mL). In NMO patients, the levels of IFN-*α* were significantly associated with EDSS; EDSS increased by 1 when IFN-*α* increased by 38% (95% CI: 9.5% 72.9%, *P* = 0.0062) (Figure 2). The clinical manifestations in the IFN-*α* positive NMO patients included LETM, ON, and brainstem syndromes and did not differ from the rest of the patients.

The IFN-*β* levels were similar in the NMO (2.6–150 pg/mL) and MS (4.4–150 pg/mL) groups. No significant association was observed between the IFN-*β* levels and EDSS neither in NMO nor in MS patients.

## 4. Discussion

In the current study, IFN-*α* was detected significantly more often in the serum of NMO patients than in that of MS patients. We observed a higher frequency of IFN-*α* in NMO patients with acute clinical attacks and high EDSS scores. Furthermore, our logistic regression analysis indicated an association of IFN-*α* levels with disease severity (EDSS). The clinical phenotype of NMO patients with IFN-*α* was not different from the rest of NMO patients with regard to clinical manifestations and MRI findings [13]. However, this subgroup of NMO patients had significantly higher disease activity and severity compared to the rest of the NMO patients and the MS disease control group. In conformity with these observations, a recent experimental study by our group identified endogenous IFN-1 signaling as a pathway controlling severity of NMO-like pathology [12]. IFNAR-deficient mice had reduced NMO-like pathology including astrocyte pathology and granulocyte infiltration compared to wild type mice [12]. These data support a role for IFN-*α* in disease perpetuation, which may explain a negative effect of IFN-1 treatment in NMO patients.

IFN-I is constitutively expressed and produced at low levels in healthy individuals. The activation of IFN-1 pathways as an important component of the innate immune system has been observed in a number of autoimmune/inflammatory diseases, either as anti-inflammatory and therapeutic in MS [20, 21] or as proinflammatory and pathogenic in SLE, primary Sjögren's syndrome, dermatomyositis, scleroderma, and type 1 diabetes mellitus [21, 22]. In 4 to 19% of patients, therapeutic administration of IFN-*α* can induce autoantibodies and autoimmune disease, including SLE [23] and autoimmune thyroiditis [24]. To a lesser extent treatment with IFN-*β* can also be accompanied by development of autoantibodies and appearance of clinical autoimmunity [25, 26]. Interestingly, it has been observed that a patient who was treated with IFN-*α* for chronic hepatitis C infection developed NMO [27]. The IFN-induced occurrence of autoantibodies and autoimmune diseases raises speculation of the possible role of IFN-1 in autoimmune disease pathogenesis [23]. Several studies have highlighted the role of endogenous IFN-*α* activation in SLE pathogenesis and reported an association between elevated levels of serum IFN-*α* and activity and severity of the disease [6, 21]. These data justified the use of SLE patient material as a positive disease control group in the present study.

NMO is a disease with autoimmune characteristics associated with immunologic abnormalities including pathogenic autoantibodies (anti-AQP4 antibody) and complement activation followed by inflammatory activity [2]. Furthermore, granulocytosis is a characteristic feature of inflammatory infiltrates in NMO that distinguishes it from MS [2, 28]. A recent study reported that serum IFN-1 activity and IFN-*β*-induced responses in PBMNC were elevated in NMO patients as opposed to MS patients [31]. Clinical trials have indicated that IFN-*β* therapy is ineffective for prevention of NMO disease activity and may even exacerbate disease [10, 11, 32]. These observations raise the question of a possible role of IFN-1 in NMO pathogenesis. Additionally, clinical and experimental data have suggested that interferon-beta nonresponders have elevated levels of endogenous IFN-1 prior to treatment [33]. In the present study, we observed a link between IFN-*α* and NMO disease activity and severity. This observation may explain negative effects of IFN-*β* therapy and suggest implications of endogenous IFN-1 in NMO. Thus, why IFN-1 signaling would be protective in MS and pathogenic in NMO likely relates to different mechanisms of diseases.

Since NMO is a severe inflammatory demyelinating disease of the CNS with a less favorable prognosis than MS [34] and with different treatment approaches, early diagnosis is critical [34]. As well as other inflammatory autoimmune diseases, immunological biomarkers may play an important role in the diagnosis of NMO. Whether IFN-*α* will qualify as a marker of inflammation for assessment of NMO diagnosis and disease activity requires further evaluation in larger, preferably longitudinal studies.

## 5. Conclusions

In conclusion, we observed detectable levels of IFN-*α* in a subgroup of NMO patients, significantly more often than in MS patients. IFN-*α* levels were associated with clinical disease activity and severity. This observation suggests a possible link between IFN-*α* and NMO and may provide a plausible explanation for a negative effect of type 1 IFN treatment in NMO as well as open new perspectives for improving diagnosis, therapy, and understanding of disease pathogenesis.

## Figures and Tables

**Table 1:** Clinical characteristics of patients with neuromyelitis optica (NMO) and multiple sclerosis (MS).

Clinical characteristics	NMO *n* = 36	MS *n* = 41	*P* value	OR (95%)
Detectable interferon-alpha	9/36	2/41	0.0197	6.5 (1.18–64.96)
Females	25	33	0.2986	0.55 (0.17–1.77)
Positive anti-AQP4 Ab	22	0	<0.0001	NA*
≥3 vertebral segments spinal cord lesion	30	0	<0.0001	NA
Treatment	27	36	0.2358	0.42 (0.01–1.59)
Interferon-beta	6	27	<0.0001	0.10 (0.03–0.34)
Natalizumab	15	9	0.0851	2.54 (0.85–7.82)
Azathioprine	5	0	0.0191	NA
Rituximab	1	0	0.4675	NA
EDSS; median (range)	5 (2–9)	4 (2–9)	0.046	NA

*NA: Nonapplicable.

**Table 2:** Clinical characteristics of neuromyelitis optica (NMO) and multiple sclerosis (MS) patients with detectable interferon-alpha.

Clinical characteristics	NMO *n* = 9	MS *n* = 2
Females	5	2
Age of onset: median (range)	36 (20–64)	27 (25–29)
Duration of disease: median (range), year	7.9 (2–20)	6.5 (2–18)
Positive anti-aquaporin-4 antibody	5	0
EDSS; median (range)	7.5 (5–9)	4.5 (4–5)
Acute relapse at investigation time	7	1
≥3 vertebral segments spinal cord lesion	9	0
Optic neuritis	9	1
Brainstem syndrome	6	1
Treatment	7	2
Interferon beta	1	1
Natalizumab	4	1
Azathioprine	1	0
Rituximab	1	0

## Abbreviations

AQP4: Aquaporin-4
EDSS: Expanded Disability Status Scale
IFN: Interferon
IFN-*α*: IFN-alpha
IFN-*β*: Interferon-beta
LETM: Longitudinally extensive transverse myelitis
MS: Multiple sclerosis
MRI: Magnetic resonance imaging
NMO: Neuromyelitis optica.
